# Serum Metabolomics Reveals Higher Levels of Polyunsaturated Fatty Acids in Lepromatous Leprosy: Potential Markers for Susceptibility and Pathogenesis

**DOI:** 10.1371/journal.pntd.0001303

**Published:** 2011-09-06

**Authors:** Reem Al-Mubarak, Jason Vander Heiden, Corey D. Broeckling, Marivic Balagon, Patrick J. Brennan, Varalakshmi D. Vissa

**Affiliations:** 1 Department of Microbiology, Immunology and Pathology, Colorado State University, Fort Collins, Colorado, United States of America; 2 Proteomics and Metabolomics Facility, Colorado State University, Fort Collins, Colorado, United States of America; 3 Leonard Wood Memorial Center for Leprosy Research, Cebu, Philippines; University of California San Diego School of Medicine, United States of America

## Abstract

**Background:**

Leprosy is a disease of the skin and peripheral nervous system caused by the obligate intracellular bacterium *Mycobacterium leprae*. The clinical presentations of leprosy are spectral, with the severity of disease determined by the balance between the cellular and humoral immune response of the host. The exact mechanisms that facilitate disease susceptibility, onset and progression to certain clinical phenotypes are presently unclear. Various studies have examined lipid metabolism in leprosy, but there has been limited work using whole metabolite profiles to distinguish the clinical forms of leprosy.

**Methodology and Principal Findings:**

In this study we adopted a metabolomics approach using high mass accuracy ultrahigh pressure liquid chromatography mass spectrometry (UPLC-MS) to investigate the circulatory biomarkers in newly diagnosed untreated leprosy patients. Sera from patients having bacterial indices (BI) below 1 or above 4 were selected, subjected to UPLC-MS, and then analyzed for biomarkers which distinguish the polar presentations of leprosy. We found significant increases in the abundance of certain polyunsaturated fatty acids (PUFAs) and phospholipids in the high-BI patients, when contrasted with the levels in the low-BI patients. In particular, the median values of arachidonic acid (2-fold increase), eicosapentaenoic acid (2.6-fold increase) and docosahexaenoic acid (1.6-fold increase) were found to be greater in the high-BI patients.

**Significance:**

Eicosapentaenoic acid and docosahexaenoic acid are known to exert anti-inflammatory properties, while arachidonic acid has been reported to have both pro- and anti-inflammatory activities. The observed increase in the levels of several lipids in high-BI patients may provide novel clues regarding the biological pathways involved in the immunomodulation of leprosy. Furthermore, these results may lead to the discovery of biomarkers that can be used to investigate susceptibility to infection, facilitate early diagnosis and monitor the progression of disease.

## Author Summary

Leprosy is an infectious disease caused by the obligate intracellular bacterium *Mycobacterium leprae*. *M. leprae* infects the skin and nerves, leading to disfigurement and nerve damage, with the severity of the disease varying widely. We believe there are multiple factors (genetic, bacterial, nutritional and environmental), which may explain the differences in clinical manifestations of the disease. We studied the metabolites in the serum of infected patients to search for specific molecules that may contribute to variations in the severity of disease seen in leprosy. We found that there were variations in levels of certain lipids in the patients with different bacterial loads. In particular, we found that three polyunsaturated fatty acids (PUFAs) involved in the inhibition of inflammation were more abundant in the serum of patients with higher bacterial loads. However, we do not know whether these PUFAs originated from the host or the bacteria. The variations in the metabolite profile that we observed provide a foundation for future research into the explanations of how leprosy causes disease.

## Introduction

Leprosy is caused by *Mycobacterium leprae*, an obligate intracellular pathogen, which infects the skin and peripheral nerves. *M. leprae* invasion of Schwann cells leads to nerve damage, disability and deformity [1]–[2]. However, not all infected patients have the same clinical course. The course of the disease may be punctuated by spontaneous and/or recurring episodes of immunological imbalances that need immediate medical attention and immune suppressive treatment. There are no routine laboratory tests for monitoring clinical improvement, response to treatment or evolution of drug resistance, aside from monitoring the reduction of bacillary levels in skin smears. Even after several decades of multidrug therapy programs to reduce leprosy transmission, incidence is not declining at expected rates in some of the most endemic countries [3]. This persistent incidence in some regions is commonly believed to be due to undetected and undiagnosed subclinical cases [4].

Leprosy is conventionally described as a spectral disease using the well-established Ridley-Jopling scheme [5]. At one pole is the limiting form termed tuberculoid (TT) leprosy. In tuberculoid leprosy the bacterial load is low due to effective cell mediated immunity (CMI), and the infection is usually localized to either a skin patch or nerve trunk. At the site of infection, the immune response is dominated by Th1 associated pro-inflammatory cytokines (IFN-γ and IL-2) and granuloma formation. The opposite profile form is lepromatous (LL) leprosy, which shows a high bacterial load, poor CMI, and is characterized by Th2 associated anti-inflammatory cytokines (IL-4 and IL-10) and antibody production. Between the poles are borderline tuberculoid (BT), borderline (BB) and borderline lepromatous (BL).

There are multiple known and undefined factors that modulate the range of susceptibility to clinical outcomes, including metabolic and immune functions. The individual contributions of host and bacterium are not yet fully defined, although many human genetic loci and bacterial components have been implicated in the process of infection and perturbation of the immune response [6]–[10]. Host factors include single nucleotide polymorphisms (SNPs) in genomic regions associated with a variety of products such as TNF-α, IL-10, vitamin D receptor (VDR), parkin (PARK2) and parkin co-regulated gene protein (PACRG) [11]–[12]. Nutritional and metabolic factors may also play a role in regulating the host immune response [13]–[14]. The pathogen *M. leprae* is unique in that its genome has undergone massive decay, particularly in catabolic pathways and energy generating processes, and is therefore thought to be highly dependent on the host system for growth [15]. Novel overlapping mechanisms have been described by which *M. leprae* modulates its environment for nutrition and immune evasion [16].

In this context, where leprosy is a product of complex host-pathogen relationships, there is a need for modern approaches to uncover underlying and/or novel biochemical signals that may be informative regarding those pathways that contribute to disease. Though leprosy is a disease of the skin and peripheral nerves, there may be biomarkers in the blood (circulatory biomarkers) which may indicate systemic factors. Several investigators have studied plasma and serum lipid composition in patients using traditional analytical methods such as thin layer chromatography (TLC) or gas chromatography (GC) [17]–[18]. With the advent of sensitive ultrahigh pressure liquid chromatography (UPLC) quadrupole time-of-flight (Q-TOF) mass spectrometry (UPLC-MS), separation and detection of large numbers of small molecules (metabolites) in complex starting mixtures has become feasible. UPLC-MS provides rapid screening with accurate mass measurement, is of high resolution, has low-detection limits, permits ion fragmentation, and does not require a large amount of sample or a combination of different techniques to identify metabolites. This technology has made it possible to rapidly identify biomarkers which distinguish normal states from various disease states using biological specimens such as urine, plasma and serum [19]–[21].

We sought to use this metabolomics approach to contrast the serum metabolome of patients with high and low bacterial indices (BI) using UPLC-MS. In the high-BI serum we discovered greater levels of the polyunsaturated fatty acids (PUFAs) eicosapentaenoic acid (EPA), arachidonic acid (AA) and docosahexaenoic acid (DHA). We discuss these findings in the context of emerging models regarding the interactions between lipid metabolism and immunity. The methods and findings have implications for discovery of novel biomarkers for diagnosis, identification of therapeutic targets and elucidation of pathogenic mechanisms.

## Materials and Methods

### Ethical Statement

Ethical approval for the use of these stored samples for research was obtained from the Institutional Review Board of Colorado State University and the Cebu Skin Clinic. Patient samples were collected following written informed consent.

### Serum Sample Collection, Preparation and Selection

Sera were selected from a sample bank generated for ongoing research into the molecular epidemiology of leprosy involving newly diagnosed leprosy patients at the Cebu Skin Clinic in Cebu, Philippines [22]. Samples were taken prior to the initiation of multidrug therapy. Blood samples were drawn into a plain (no additive) evacuated tube (BD Vacutainer Serum) and centrifuged at 1,500 rpm for 10 min at 4°C in a refrigerated centrifuge. The serum samples were aliquoted into multiple vials at 1 ml per vial and frozen at −20°C until shipment. The sera were shipped on dry ice to Colorado State University and stored at −20°C until subsequent use in the laboratory.

Serum samples were selected from two groups of patients, those with BI<1 (n = 10) and those with BI>4 (n = 13) (**Table 1**). Sample selection was randomized and without consideration of clinical or demographic data aside from BI. Though factors such as age, gender, clinical presentation and medical history were not considered in the study design or analysis, such data were collected during patient intake and are presented in **Table 1**. BI was measured at the Cebu Skin Clinic by microscopic examination of acid-fast stained slit-skin smears taken from six sites, including representative active lesions. BI was ranked on a log 10 scale from 0 to 6 [23].

**Table 1:** Patient demographic and clinical data.

Sample^a^	BI^b^	R-J Class^c^	PB/MB^d^	Sex	Age	Duration of Symptoms^e^	Medical History^f^
**Low-BI**							
L5	0.33	BT	MB	F	20	2 Y	-
L32^g^	0.17	BT	MB	M	27	1 Y	-
L40^g^	0.50	-	MB	M	38	-	-
L49	0.00	BT	PB	M	36	2 Y	-
L74	0.33	BT	MB	M	62	5 M	Hypertension
L76	0.50	BL	MB	M	64	5 M	-
L77	0.00	BT	PB	M	36	10 Y	-
L79	0.00	BT	PB	F	64	2 Y	-
L85	0.17	BL	MB	F	42	5 M	-
L90	0.00	BT	MB	F	24	2 Y	-
**High-BI**							
L1	4.80	LL	MB	M	26	2 Y	-
L9	4.70	LL	MB	M	25	5 Y	-
L11	4.80	LL	MB	F	22	15 Y	Congenital deformities
L15	5.00	LL	MB	M	25	2 Y	-
L19	4.80	LL	MB	M	28	6 Y	Appendicitis
L22	5.00	LL	MB	M	41	3 Y	Peptic Ulcer
L29	4.80	LL	MB	M	18	3 Y	-
L51	5.00	LL	MB	M	61	5 Y	-
L53^h^	5.00	LL	MB	M	39	4 Y	-
L58	5.00	LL	MB	M	37	2 Y	-
L75	5.00	LL	MB	M	26	2 Y	-
L88	5.00	LL	MB	M	49	3 Y	-
L89	5.00	LL	MB	M	30	1 Y	-

a Sample names are as per Sakamuri et al, 2009 [22].

b Bacterial index determined from slit-skin smear [23].

c Ridley-Jopling classification of leprosy [5].

d Clinical classification into either paucibacillary (PB) or multibacillary (MB) leprosy.

e Self-reported duration of symptoms prior to treatment.

f Self-reported medical history; reported conditions do not reflect current illness at the time of diagnosis with leprosy.

g Patients who presented at the clinic in a type 1 reaction state.

h Patients with deformities caused by leprosy.

A volume of 50 µl from each serum sample was prepared for analysis by UPLC-MS. Sera proteins were precipitated by the addition of 3 volumes (150 µl) of cold 100% methanol. The samples were vortexed, placed at −20°C for two hours, then centrifuged for 10 minutes at 15,000 rpm to pellet the protein precipitate. The supernatants were carefully transferred to new Eppendorf tubes. From each supernatant, 1 µl was analyzed by UPLC-MS in both negative and positive modes with duplicate injections. To confirm the observations and mass spectrometry methods a subset of the selected sera were reanalyzed using new aliquots and triplicate injections. Five each from the low-BI (L32, L40, L76, L79, L85) and high-BI (L1, L11, L19, L58, L88) groups were pooled and retested; two low-BI (L40, L85) and two high-BI (L15, L88) samples were retested individually.

### Instrumentation and UPLC-MS Methods

The serum methanol extracts were separated on a Waters ACQUITY UPLC coupled with a Q-TOF under the control of MassLynx v4.1 [Waters. Millford, MA, USA]. Sample injections (1 µl) were performed on a Waters ACQUITY UPLC system. Separation was performed using a Waters ACQUITY UPLC C8 column (1.7 µM, 1.0×100 mm), using a gradient from solvent A (89% water, 5% acetonitrile, 5% isopropanol, 1% 500 mM ammonium acetate) to solvent B (49.5% acetonitrile, 49.5% isopropanol, 1% 500 mM ammonium formate). Injections were made in 100% A, which was held for 0.1 min. A 0.9 min linear gradient to 40% B was applied, followed by a 10 min gradient to 100% B which was held for 3 min, then returned to starting conditions over 0.1 min, and then allowed to re-equilibrate for 5.9 min. Flow rate was constant at140 µl/min for the duration of the run. The column was held at 50°C; samples were held at 5°C.

Mass data were collected between 50 and 1200 m/z at a rate of two scans per second. The voltage and temperature parameters were as follows: 3000 V capillary, 30 V sample cone, 2.0 V extraction cone, 350°C desolvation temperature and 130°C source temperature. Calibration was performed prior to sample analysis via infusion of sodium formate solution, with mass accuracy within 5 ppm. For MS/MS, the parent ion was selected by quadrupole and fragmented via collision-induced dissociation (CID) with argon at collision energy of 20 eV for fatty acids and 30 eV for phospholipids.

### Data Processing

UPLC-MS data were aligned, extracted and viewed using MarkerLynx v4.1 [Waters. Millford, MA, USA]. Chromatographic peaks were detected between 0 and 28 min with a retention time (RT) window of 0.1 min. Apex track peak detection parameters were used, with automatic detection of peak width and baseline noise. The spectrometric features were assigned by m/z and RT, while the relative intensity was based on the area of all features. Initial screening for compounds with significant differences in abundance between the low-BI and high-BI groups was performed by orthogonal projection onto latent structures (OPLS) with the software SIMCA-P+ v12.0 [Umetrics. Umeå, Västerbotten, Sweden], using a Po(corr) cut-off of 0.5. Further statistical analysis was performed using several R packages within R 2.12.1 [24]. Principal component analysis (PCA) was performed using the package stats::prcomp with both scaling and centering of the variables. Generation of receiver operating characteristic (ROC) curves for selected compounds was performed using the R package pROC v1.4.3 [25]; a 95% confidence interval was generated for sensitivity using 2,000 bootstrap replicates. For each selected compound, histogram bins were calculated using the Freedman-Diaconis rule and kernel density estimates were calculated using Gaussian smoothing. In order to compare the first (individual serum) and second (pooled sera) runs, data for each compound was standardized by subtracting the mean and dividing the result by the standard deviation (standard score) to account for shifts in instrument sensitivity over time; the data were then compared for statistically significant differences using the Mann-Whitney test.

### Identification of Compounds

Tentative compound class assignments (free fatty acid, glycerolipid, phospholipid, etc.) were made by querying the exact mass against the LIPID MAPS database [26] and the online web server MassTRIX: Mass Translator into Pathways [27]. The compounds that showed significant differences in intensity between the low-BI and high-BI groups, based on exact m/z and 0.05 min RT differences, were further fragmented by MS/MS in both positive and negative ion modes. Metabolite identities were manually examined for signature ions and verified by comparing the fragment spectra to those in LIPID MAPS and published data [28]. MassTRIX was also used to explore related pathways that may be associated with selected metabolites.

### Standards

After preliminary assignments were made for some of the selected compounds, pure standards were obtained and analyzed by the previously described chromatographic methods. Pooled sera were rerun along side the standards. Eicosapentaenoic acid (EPA, 20∶5), arachidonic acid (AA, 20∶4) and docosahexaenoic acid (DHA, 22∶6) were purchased from Sigma-Aldrich [Saint Louis, MO, USA]; 1-palmitoyl-2-arachidonoyl-*sn*-glycero-3-phosphocholine (PAPC) was purchased from Avanti Polar Lipids [Alabaster, Alabama, USA]. All standards were dissolved in 75% methanol prior to UPLC-MS analysis.

## Results

### Global Characterization of Mass Spectrometry Data

**Figure 1:**
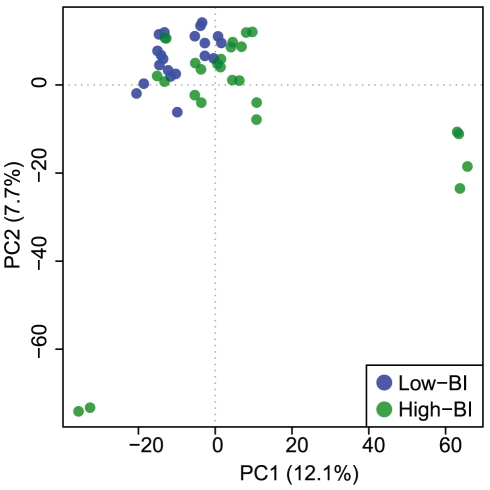
Principal component analysis of all positive and negative mode m/z values detected in serum of leprosy patients. A PCA score plot of all positive mode (n = 1668) and negative mode (n = 2489) m/z values collected from UPLC-MS analysis of 23 serum samples (10 low-BI, 13 high-BI). The first two components account for 20.4% of the variation in the data. Duplicate runs of each sample are visible as clustered pairs. A separation of samples is seen based on the BI of the patient.

The UPLC-MS data were first characterized globally. Across both the low-BI (n = 10) and high-BI (n = 13) samples a total of 1668 features in the positive mode and 2489 features in negative mode were observed (**Supplement S1**). A PCA, generated from abundance data of all positive and negative mode m/z-RT pairs (features), showed low-BI and high-BI patient sera clustering away from each other (**Figure 1**). The separation of patient groups indicates that there are m/z-RT pairs that are quantitatively distinct in the two groups. Close clustering of injection duplicates is also seen, which is the expected behavior.

### Selection and Validation of Metabolite Biomarkers

To identify the compounds that distinguished the low-BI from high-BI samples, the dataset was first pared down to features which exhibited the greatest difference in abundance between the two sample groups with OPLS (not shown). This yielded 48 features with masses up to approximately 1 kDa: 19 from the positive mode and 29 from the negative mode. From these 48 features, 18 compounds were tentatively identified using the online databases LIPID MAPS and MassTRIX (**Table 2**). The data indicate an increase in the level of several lipids in the high-BI sera compared to those in the low-BI sera. All of the 18 identified compounds were more abundant in the high-BI samples except for those with m/z 518.3245, 558.3196 and 566.3192, which were more abundant in the low-BI samples. A confirmatory second UPLC-MS analysis was performed using a pooled subset of samples. Though m/z and RT values shifted slightly, due to expected operational variability, we found the same 18 compounds again showed quantitative distinctions between low-BI and high-BI groups.

**Table 2:** Identities of significant features, observed values and measures of statistical support.

							Median Abundance^e^		
m/z^a^	RT^b^	Mode^c^	Name	Formula	Identified By^d^	LIPID MAPS ID	Low-BI	High-BI	AUC^f^
**Identified Compounds (1)** h									
301.2174^g^	3.8001	−	Eicosapentaenoic Acid (EPA) (20∶5)	C_20_H_30_O_2_	Standard+LM MS/MS	LMFA01030759	56.5	151.6	86.2±11.2
303.2324^g^	4.2763	−	Arachidonic Acid (AA) (20∶4)	C_20_H_32_O_2_	Standard+LM MS/MS	LMFA01030001	383.5	780.9	91.9±15.3
327.2340^g^	4.1138	−	Docosahexaenoic Acid (DHA) (22∶6)	C_22_H_32_O_2_	Standard+LM MS/MS	LMFA01030185	238.2	474.3	82.7±12.7
**Putatively Annotated Compounds (2)** h									
317.2114^g^	2.6914	−	5-oxo-eicosatetraenoic Acid (5-oxoETE)	C_20_H_30_O_3_	LM MS/MS	LMFA03060011	22.0	46.9	71.5±15.0
329.2465^g^	4.4796	−	Docosapentaenoic Acid (DPA) (22∶5)	C_22_H_34_O_2_	LM MS/MS	LMFA01030183	36.8	113.3	90.2±9.4
335.2234^g^	4.0446	−	Leukotriene B4 (LTB4)	C_20_H_32_O_4_	LM MS/MS	LMFA03020001	3.5	7.3	72.1±15.5
516.3074^g^	3.7331	+	Lyso PC (18∶4/0∶0)	C_26_H_46_NO_7_P	LM MS/MS	LMGP01050040	18.8	31.2	87.9±10.0
**Unconfirmed or Unknown Compounds (3,4)** h									
279.2317	2.8074	+	γ-Linolenic Acid (18∶3)	C_18_H_30_O_2_	MassTRIX	LMFA01030141	2.2	5.5	74.8±14.9
			or α-Linolenic Acid (18∶3)	C_18_H_30_O_2_	MassTRIX	LMFA01030152			
283.2423	4.1115	−	Stearic Acid (18∶0)	C_18_H_36_O_2_	MassTRIX	LMFA01010018	71.5	135.3	81.9±13.0
305.2500	4.6343	−	Eicosatrienoic Acid (ETrE) (20∶3)	C_20_H_34_O_2_	LM Mass	LMFA01030157	73.0	151.5	87.7±10.6
379.2847	5.4213	+	-	-	-	-	4.2	14.2	91.9+8.1
385.2387	4.2658	−	-	-	-	-	3.5	5.2	82.2±12.2
395.2240	4.1131	−	Echitovenine	C_23_H_28_N_2_O_4_	MassTRIX	-	1.3	3.8	85.8±11.0
509.3366	3.2629	+	Lyso PC (O-18∶1/0∶0)	C26H_55_NO_6_P	LM Mass	LMGP01070009	0.0	5.2	97.0±5.7
			or Lyso PE (20∶1/0∶0)	C_25_H_51_NO_7_P	Murphy	-			
518.3245	2.7152	+	Lyso PC (18∶3/0∶0)	C_26_H_48_NO_7_P	LM Mass+Murphy	LMGP01050038	34.0	17.7	84.8±12.3
558.3196^g^	2.7183	+	Lyso PC + unknown fatty acid	-	-	-	10.8	5.7	86.9±12.4
566.3192	2.8304	+	Lyso PC (21∶0/0∶0)	C_29_H_60_NO_7_P	LM Mass	LMGP01050051	4.1	0.0	75.4±12.2
798.5707^g^	4.2907	−	PAPC-like	-	Standard+LM MS/MS	LMGP01010007 (PAPC)	11.0	23.2	79.8±12.8

a The observed mass to charge ratio.

b The observed retention time.

c Whether the m/z-RT pair was observed in position (+) or negative (−) ion mode.

d The basis for the identification of the compound. Standard: Comparison of MS/MS spectrum and RT to a commercial standard. LM MS/MS: Comparison of MS/MS spectrum to spectra published in LIPID MAPS. MassTRIX: Identified by MassTRIX based upon exact mass. LM Mass: Compound identity based upon exact mass matches in LIPID MAPS. Murphy: The compound was compared to mass data in Murphy, 2002 [30].

e The median abundance value observed in the individual (non-pooled) samples.

f The area under the curve (AUC) of the receiver operating characteristic (ROC) curve with a 95% confidence interval denoted as a ± value.

g Compounds which fragmented in MS/MS. See Figures 2, 3, 4 and Supplement S2 for MS/MS spectra of samples and commercial standards. See Supplement S3 for additional spectra.

h Confidence levels for compound identifications as per Sumner et al, 2007 [31]. Lower values indicate a more confident identification.

We also queried the complete list of m/z values against the MassTRIX annotation system, which performs a search for potential compound identities and associated pathways curated in KEGG: Kyoto Encyclopedia of Genes and Genomes [29]. MassTRIX assigned a total of 74 negative mode features to 143 compounds in 40 pathways, and 79 positive mode features to 89 compounds in 51 pathways. The predominant hits were pathways involved in AA metabolism (29 compounds) and synthesis of unsaturated fatty acids (13 compounds); not all compounds were unique.

### Compound Identification by Tandem Mass Spectrometry

The 18 significant compounds that we tentatively identified were further characterized by MS/MS. From these 18 compounds, the compounds of the most interest to us - given their role in modulation of the inflammatory response - were the n-6 PUFA AA, the n-3 PUFAs EPA and DHA, and the compound with structural similarity to PAPC. Commercial standards of EPA, AA, DHA and PAPC were obtained and submitted to mass spectrometry in parallel with the serum samples. Not all of the 18 ions fragmented, but compound confirmation was achieved via MS/MS for 9 compounds by referencing the ion fragmentation pattern against published spectra and/or available standards (**Table 2**) [30]–[31].

The chemical structures and fragmentation patterns of compounds listed in **Table 2** are shown in **Figures 2** **,** **3** **,** **4** and **Supplements S2, S3**. In each of **Figures 2** **,** **3** **,** **4** and **Supplement S2** the chemical structure of the compound is shown in **Panel A**, the fragmentation pattern of the commercial standard is shown in **Panel B**, and the fragmentation pattern of the corresponding compound in the pooled serum sample is shown in **Panel C. Supplement S3** shows the fragmentation pattern in the pooled serum sample for the remaining compounds putatively identified by MS/MS. The spectra illustrations have been adjusted from the original MassLynx output files for clarity; the font of the axes and labels has been changed, the line width of the spectra has been increased, and extraneous text and borders have been removed.

**Figure 2:**
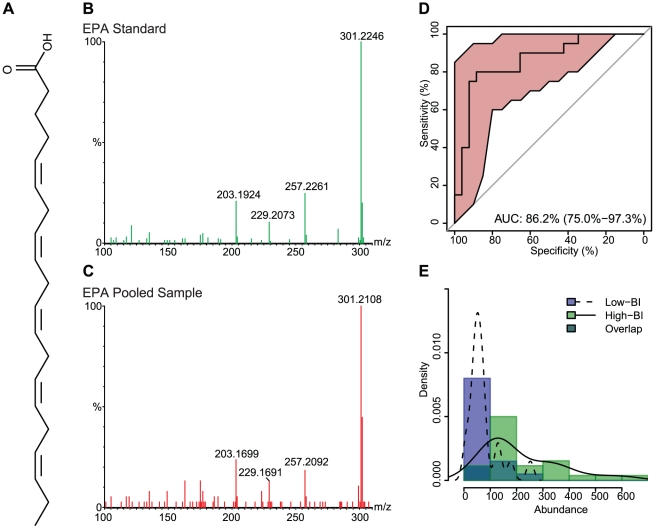
Eicosapentaenoic acid (EPA) chemical structure, MS/MS spectra, ROC curve and distribution across sample groups. (**A**) The chemical structure of EPA. (**B**) The MS/MS fragmentation pattern for the commercial standard. (**C**) The MS/MS fragmentation pattern of a representative pooled serum sample. (**D**) An ROC curve, showing the diagnostic accuracy of EPA in distinguishing low-BI from high-BI samples. The shaded (red) region surrounding the curve represents a 95% confidence interval for sensitivity. The AUC is shown on the graph with a 95% confidence interval in parenthesis. (**E**) A histogram showing the distribution of EPA in the low-BI and high-BI groups. The overlaid curves show the kernel density estimates for each sample group.

**Figure 3:**
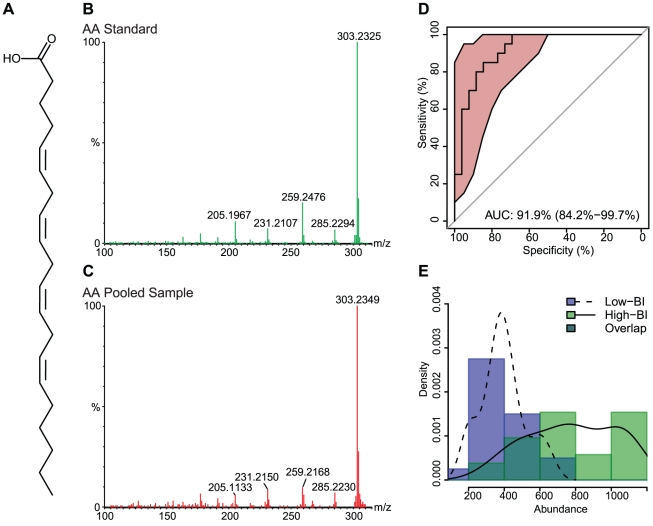
Arachidonic acid (AA) chemical structure, MS/MS spectra, ROC curve and distribution across sample groups. (**A**) The chemical structure of AA. (**B**) The MS/MS fragmentation pattern for the commercial standard. (**C**) The MS/MS fragmentation pattern of a representative pooled serum sample. (**D**) An ROC curve, showing the diagnostic accuracy of AA in distinguishing low-BI from high-BI samples. The shaded (red) region surrounding the curve represents a 95% confidence interval for sensitivity. The AUC is shown on the graph with a 95% confidence interval in parenthesis. (**E**) A histogram showing the distribution of AA in the low-BI and high-BI groups. The overlaid curves show the kernel density estimates for each sample group.

**Figure 4:**
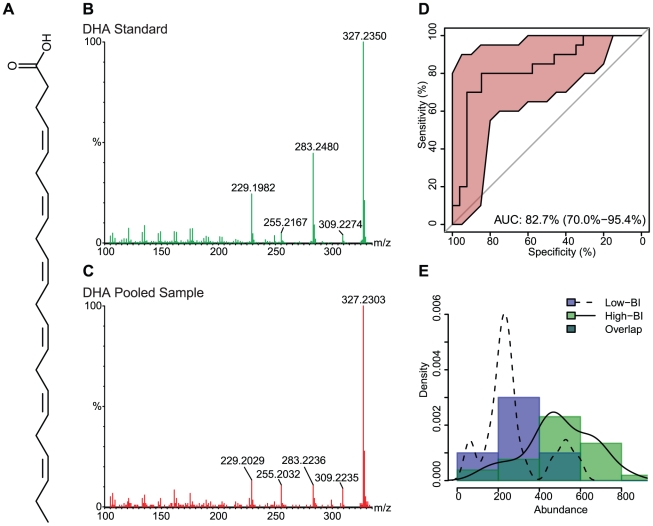
Docosahexaenoic acid (DHA) chemical structure, MS/MS spectra, ROC curve and distribution across sample groups. (**A**) The chemical structure of DHA. (**B**) The MS/MS fragmentation pattern for the commercial standard. (**C**) The MS/MS fragmentation pattern of a representative pooled serum sample. (**D**) An ROC curve, showing the diagnostic accuracy of DHA in distinguishing low-BI from high-BI samples. The shaded (red) region surrounding the curve represents a 95% confidence interval for sensitivity. The AUC is shown on the graph with a 95% confidence interval in parenthesis. (**E**) A histogram showing the distribution of DHA in the low-BI and high-BI groups. The overlaid curves show the kernel density estimates for each sample group.

The parent and daughter ions of EPA, AA and DHA appear as expected in both the standards and patient samples. However, we could not conclusively identify the feature we observed in the serum samples with m/z of 798. The molecular weight of PAPC is 781, with an observed value of 766 under negative ionization due to loss of the methyl group from choline (**Supplement S2B**). The feature with m/z of 798 produced several fragments consistent with the PAPC standard; specifically, ions with m/z 255, 303 and 480 (**Supplement S2C**), which correspond to palmitic acid, arachidonic acid and lysophosphocholine (16∶0/0∶0), respectively. The RT for the PAPC standard was 8.5 min, while the RT for the feature with m/z 798 was 4.3 min. It is possible, but not confirmed, that the observed feature with an m/z of 798 is an oxidized form of PAPC [32]; additional investigation was performed, but did not yield satisfactory results.

### Statistical Support for Biomarkers

The diagnostic accuracy of each feature, as measured by the extent to which each feature accurately distinguishes low-BI from high-BI samples, was determined using receiver operating characteristic (ROC) curves [33]. The ROC curve for the feature compares the distribution of abundance between low-BI and high-BI samples. The more the curve is pulled toward the upper-left corner [higher sensitivity, higher specificity and higher area under the curve (AUC)] the less overlap between the distributions in each group, and thus the more effective the feature is at discriminating low-BI from high-BI sera. The AUC for each significant feature, along with a 95% confidence interval indicated as a ± value, is shown in **Table 2**. ROC curves for features of interest are shown in **Panel D** of **Figures 2** **,** **3** **,** **4** and **Supplement S2**.

The distribution of abundance values of the individual samples (first experiment) can be seen in **Panel E** of **Figures 2** **,** **3** **,** **4** and **Supplement S2**, as both a histogram and kernel density estimate. In addition to comparing the abundance values across patient groups, we also compared the first (individual sample) and second (pooled sample) experiments for statistically significant differences. Although the same qualitative differences were seen across patient groups in both experiments, the two experiments showed marked differences in mean abundance values, which we believe is due to variation in instrument sensitivity between runs. The distribution of abundance values between experiments was compared using the Mann-Whitney test following standardization to account for variation between runs. Between the two experiments, the distributions for EPA (low-BI p = 0.15, high-BI p = 0.07; **Figure 2E**), AA (low-BI p = 0.65, high-BI p = 0.34; **Figure 3E**), DHA (low-BI p = 0.83, high-BI p = 0.53; **Figure 4E**) and the PAPC-like compound (low-BI p = 0.62, high-BI p = 0.38; **Supplement S2E**) were not found to differ significantly at 95% confidence.

We note that there is a sampling bias with regards to both age and sex in the selected patients (**Table 1**). Specifically, the median age is 37 in the low-BI and 28 in the high-BI, and the ratio of male to female is 6∶4 in the low-BI and 12∶1 in the high-BI. The parent study from which these patients were randomly selected (n = 310) shows a concordant bias. In the parent study, the median age of a low-BI patient (n = 63) is 37 and the median age of a high-BI patient (n = 123) is 29. The odds of selecting a male patient from the low-BI group are 37∶26 (1.42), and the odds of selecting a male patient from the high-BI group are 111∶12 (9.25). Though patient age and sex were not considered in the study design or the analysis as a whole, we investigated the diagnostic accuracy of the features listed in **Table 2** when considering only the male patients. Though some shifts were seen in the AUC and median abundance in the two BI groups, the same set of features still showed statistically significant differences between the low-BI and high-BI groups (data not shown).

## Discussion

The goal of our research was to explore the applicability of non-targeted metabolomics to the study of leprosy. Most research aimed at understanding variations in clinical presentations have been studies of gene expression profiles and immune response mechanisms using a variety of assays on whole blood, serum, plasma, peripheral blood mononuclear cells or skin biopsies [17], [34]–[36]. To date, metabolite profiles in leprosy have only been explored using target-based assays of blood samples [37]–[38]. These techniques are limited in terms of sample throughput, the ability to resolve individual metabolites in complex specimens, the sensitivity of feature detection, and the accuracy of compound identification. By using a metabolomics approach based on mass spectrometry, we were able to discover several metabolites in serum with differential levels in low-BI and high-BI patient groups. In particular, we found that in the high-BI group there was a statistically significant increase in abundance of the n-3 PUFAs EPA and DHA, and the n-6 PUFA AA. The identification of differential levels of PUFAs in high-BI patients is intriguing, as lipid metabolism and lipid mediators have been implicated in many disease models, both infectious and non-infectious.

It has been widely thought that n-3 PUFAs (DHA and EPA) are beneficial to human health, because of their association with mitigation of the inflammatory response in conditions such as autoimmune disorders, heart disease, arthritis and graft-versus-host disease [13], [39]–[40]. Conversely, the n-6 PUFAs (including AA) are generally considered deleterious in chronic diseases because they exert pro-inflammatory effects [39]. Ironically, it is this pro-inflammatory property that would provide the necessary anti-microbial activity to combat bacterial infections.

However, new research indicates that this is only a generalized model for the properties of the n-6 versus n-3 PUFAs. Consensus is absent on their strict pro- versus anti-inflammatory phenotypes, due to their interconnected metabolic pathways and the production of downstream products (eicosanoids). Recent studies have pointed to the benefits of AA and derived eicosanoids, finding that they had both pro- and anti-inflammatory roles. Deckelbaum and Calder found that prostaglandin E2 (PGE2) may inhibit the production of pro-inflammatory cytokines (TNF-α and IL-1) from monocytes and macrophages [41]. They also found that PGE2 inhibits production of leukotrienes (LTs), through control of 5-lipoxygenese, and induces production of lipoxins, through 15-lipooxygenase; leading to anti-inflammatory and pro-resolution activities by the action of lipoxins [41]. Based on these results, AA n-6 PUFAs may control the inflammatory response by regulating both the pro- and anti-inflammatory cytokine networks. It has also been suggested that both n-3 and n-6 PUFAs play an anti-inflammatory role due to inactivation of reactive oxygen species by the unsaturated double bond. Furthermore, PUFAs may bind to peroxisome proliferator activated receptors, thus interfering with signaling molecules such as NF-κB, and repressing transcription of a variety of genes [42]. Zeyda et al found that both n-3 and n-6 PUFAs inhibit cytokine production (TNF-α and IL-12), T cell stimulation and dendritic cell differentiation at the gene level. PUFA treated dendritic cells were shown to be associated with altered membrane lipid composition, specifically an increase in unsaturated lipids, which implicates AA and EPA as anti-inflammatory mediators [14]. In the mycobacterial disease models, enrichment of n-3 PUFAs enhances susceptibility to *Mycobacterium tuberculosis* infection *in vitro* (infected macrophages) [43]–[44]. Anes et al showed that the pro-inflammatory effect of AA promotes increased bacteria killing inside macrophages by stimulating phagosomal actin assembly. In contrast, the same authors also showed that EPA and DHA promote bacterial survival and growth inside macrophages by lowering the levels of pro-inflammatory cytokines (IFN-γ, TNF-α, IL-1 and IL-6), weakening the oxidative response and hindering phagosome maturation [45].

In leprosy, Cruz et al postulated that the fatty acids and phospholipids which accumulate in lepromatous lesions are of host origin. They found a pronounced upregulation of host genes involved in lipid metabolism, such as phospholipase A2 (PLA2) and phospholipase C (PLC), for which functional counterparts are not encoded in the *M. leprae* genome [16]. An increase in phospholipase activity may contribute to the increased serum levels of AA we observed in our high-BI patients; PLA2 catalyzes the hydrolysis of phospholipids to release arachidonate in a single-step reaction, and PLC generates diacylglycerols, from which AA can be subsequently released by diacylglycerol- and monoacylglycerol-lipases.

Several other metabolites which modulate immunity either for or against mycobacterial survival have been described in the literature. These include cholesterol (HDL or LDL derived), triglycerides and vitamin D [17]–[18], [46]–[47]. We did not observe differential levels of these metabolites in our patient groups, though this does not imply variations were not present. The nature of the starting sample and the fractionation conditions may affect the metabolite pools; this study was based only on a simple one-step methanol extraction followed by C8 reverse phase UPLC-MS. Lysophosphatidylcholines (Lyso PCs) have been shown to have a potential role in immunomodulation, particularly pro-inflammatory functions [48]. We tentatively assigned some significant features as Lyso PCs - three of which were more abundant in the low-BI sera (**Table 2**). However, these identifications are preliminary and unconfirmed at his stage.

In this study we focused solely on identifying compounds with differential levels in patient sera based on the quantitative criterion of BI, rather than the more qualitative Ridley-Jopling and paucibacillary/multibacillary systems which are not always consistent across clinics [49]. Sex was not a controlled factor in our study. Though our results do not specifically indicate whether the serum signatures we found can be explained by sex differences, there is an inherent sex bias in leprosy [3], as also evidenced in our sample sets. Further investigations which delve into whether there are specific metabolites that differentiate leprosy patients based upon other classification criteria, such clinical presentation, sex, age or genetics, would provide valuable insight into the intrinsic biological factors that contribute to bacterial growth in leprosy.

The metabolomic fingerprint we identified - higher levels of AA, DHA and EPA in the sera of high-BI leprosy patients - is consistent with diminished host innate immunity to infection [16]; reaffirming the role of altered host lipid metabolism in infection and immunity. The increased serum levels of n-3 and n-6 PUFAs we identified in high-BI patients may promote *M. leprae* survival through inhibition of both the innate and adaptive immune response of the host. These novel preliminary findings lend themselves to pathway specific genome expression analysis and further characterization of the AA derived lipid mediators. For instance, the leukotriene A4 hydrolase (*lta4h*) gene has been implicated as a susceptibility locus in leprosy and tuberculosis [50]. It is thought that the fine balance of lipoxin B4 and leukotriene B4 controls the propensity to infection or immunity. Of the 9 compounds we identified by MS/MS, those with m/z 317 and 335 are candidate AA derivatives suitable for further analysis. A longitudinal study that employs a metabolomics approach may shed light on the origins and dynamics of the lipid profile. By collecting and analyzing sera before multidrug therapy, during treatment, at the onset of reaction states and after the patient is released, we may discover fluctuations in the lipid profile over the course of the infection; enabling the ultimate aim of improving diagnostics, treatment options and creating a deeper understanding of the pathogenesis of leprosy.

## Supporting Information

Supplement S1 **Complete table of all features observed in positive and negative mode UPLC-MS.** A total of 1668 features in the positive mode and 2489 features in negative mode are listed for the individual (non-pooled) sample set. RT, m/z and integrated peak intensities are shown for each feature. Sample injection duplicates are identified after the sample name (N1, N2 or P1, P2).
(XLS)

Supplement S2 **1-palmitoyl-2-arachidonoyl-sn-phosphatidylcholine (PAPC) chemical structure, MS/MS spectra, ROC curve and distribution across sample groups.** (**A**) The chemical structure of PAPC. (**B**) The MS/MS fragmentation pattern for the PAPC commercial standard. (**C**) The MS/MS fragmentation pattern for compound structurally similar to PAPC from a representative pooled serum sample. (**D**) An ROC curve, showing the diagnostic accuracy of the PAPC-like compound in distinguishing low-BI from high-BI samples. The shaded (red) region surrounding the curve represents a 95% confidence interval for sensitivity. The AUC is shown on the graph with a 95% confidence interval in parenthesis. (**E**) A histogram showing the distribution of the PAPC-like compound in the low-BI and high-BI groups. The overlaid curves show the kernel density estimates for each sample group.
(PDF)

Supplement S3 **Spectra for additional compounds identified by MS/MS.** (**A**) The MS/MS fragmentation pattern for the compound with m/z 317.21. (**B**) The MS/MS fragmentation pattern for the compound with m/z 329.25. (**C**) The MS/MS fragmentation pattern for the compound with m/z 335.22. (**D**) The MS/MS fragmentation pattern for the compound with m/z 516.31. (**E**) The MS/MS fragmentation pattern for the compound with m/z 558.32.
(PDF)
