# Supplementary figures and images for: Serum Metabolomics Reveals Higher Levels of Polyunsaturated Fatty Acids in Lepromatous Leprosy: Potential Markers for Susceptibility and Pathogenesis
Source: PLoS Negl Trop Dis. 2011 Sep 6;5(9):e1303. doi: 10.1371/journal.pntd.0001303 (PMC3167790; doi:10.1371/journal.pntd.0001303)

**A**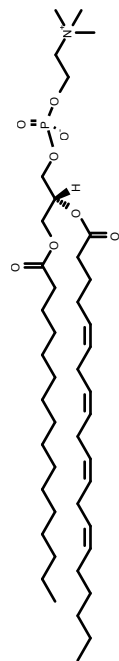**B**

PAPC Standard

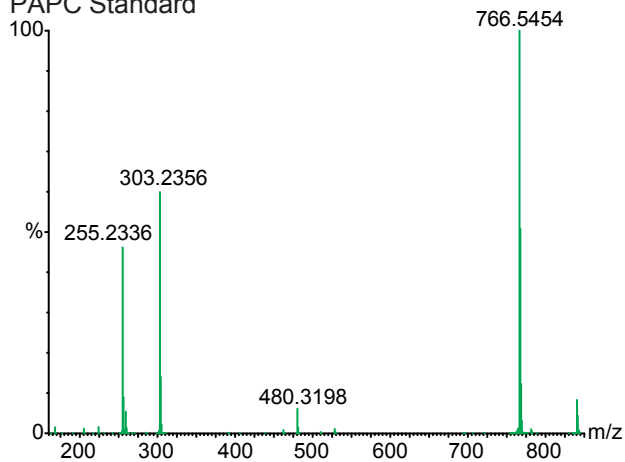**C**

PAPC-like Pooled Sample

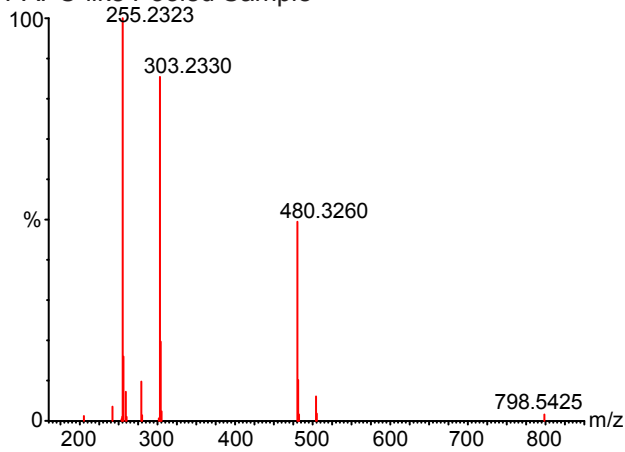**D**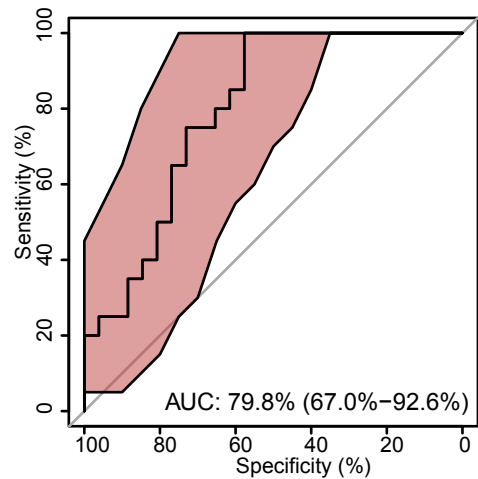**E**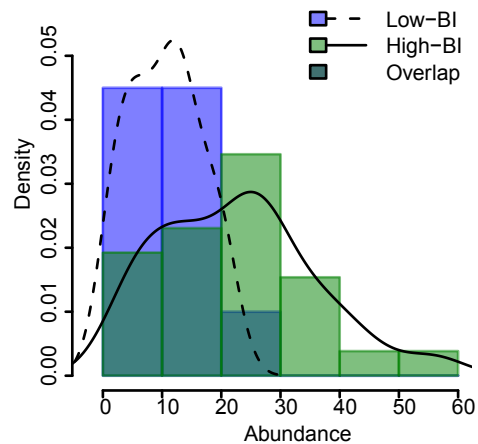

Supplement: Supplement S2 — 1-palmitoyl-2-arachidonoyl-sn-phosphatidylcholine (PAPC) chemical structure, MS/MS spectra, ROC curve and distribution across sample groups. (A) The chemical structure of PAPC. (B) The MS/MS fragmentation pattern for the PAPC commercial standard. (C) The MS/MS fragmentation pattern for compound structurally similar to PAPC from a representative pooled serum sample. (D) An ROC curve, showing the diagnostic accuracy of the PAPC-like compound in distinguishing low-BI from high-BI samples. The shaded (red) region surrounding the curve represents a 95% confidence interval for sensitivity. The AUC is shown on the graph with a 95% confidence interval in parenthesis. (E) A histogram showing the distribution of the PAPC-like compound in the low-BI and high-BI groups. The overlaid curves show the kernel density estimates for each sample group. (PDF) [file pntd.0001303.s002.pdf]

**A**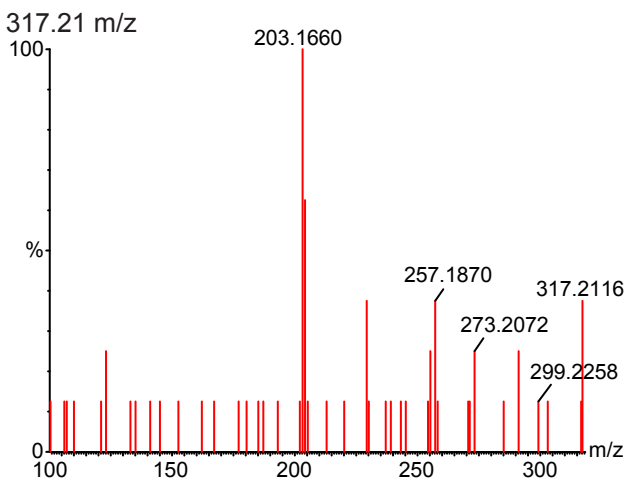**B**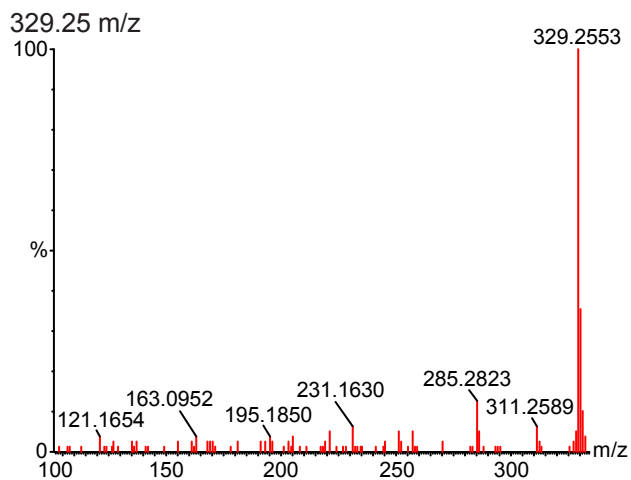**C**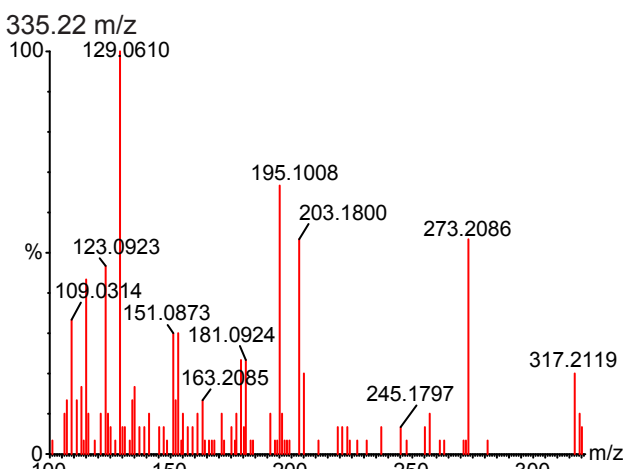**D**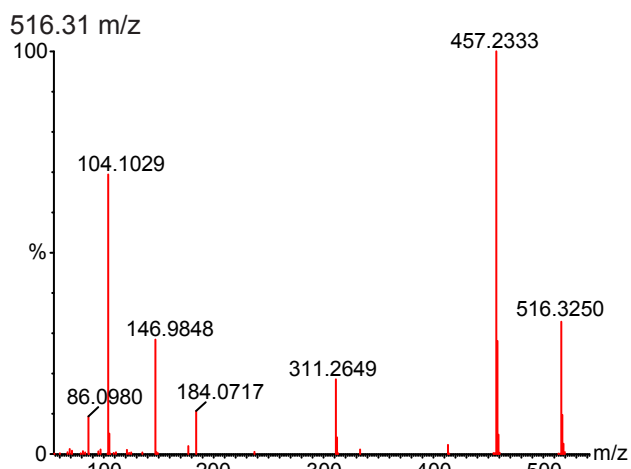**E**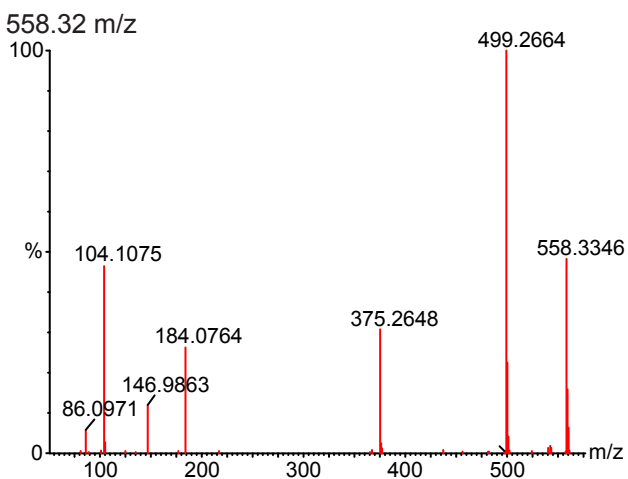

Supplement: Supplement S3 — Spectra for additional compounds identified by MS/MS. (A) The MS/MS fragmentation pattern for the compound with m/z 317.21. (B) The MS/MS fragmentation pattern for the compound with m/z 329.25. (C) The MS/MS fragmentation pattern for the compound with m/z 335.22. (D) The MS/MS fragmentation pattern for the compound with m/z 516.31. (E) The MS/MS fragmentation pattern for the compound with m/z 558.32. (PDF) [file pntd.0001303.s003.pdf]
